# Differences in genome characters and cell tropisms between two chikungunya isolates of Asian lineage and Indian Ocean lineage

**DOI:** 10.1186/s12985-018-1024-5

**Published:** 2018-08-20

**Authors:** Xiaomin Zhang, Yalan Huang, Miao Wang, Fan Yang, Chunli Wu, Dana Huang, Linghong Xiong, Chengsong Wan, Jinquan Cheng, Renli Zhang

**Affiliations:** 1 Shenzhen Center for Disease Control and Prevention, Shenzhen, 518055 China; 2 School of Public Health and Tropical Medicine, Southern Medical University, Guangzhou, 510515 China

**Keywords:** Chikungunya virus, Genome characters, Cell tropisms, Asian lineage, Indian Ocean lineage

## Abstract

**Background:**

Chikungunya virus (CHIKV) is a mosquito-transmitted *alphavirus* within the family *Togaviridae*, which has attracted global attention due to its recent re-emergence. In one of our previous studies, we successfully isolated two CHIKV virus strains, SZ1050 and SZ1239, from the serum samples of two imported patients in 2010 and 2012, respectively. However, the differences in their genome characters and cell tropisms remain undefined.

**Methods:**

We extracted the RNA of two CHIKV isolates and performed PCR to determine the sequence of the whole viral genomes. The genotypes were classified by phylogenetic analysis using the Mega 6.0 software. Furthermore, the cell tropisms of the two CHIKV isolates were evaluated in 13 cell lines.

**Results:**

The lengths of the whole genomes for SZ1050 and SZ1239 were 11,844 nt and 12,000 nt, respectively. Phylogenetic analysis indicated that SZ1050 belonged to the Indian Ocean lineage (IOL), while SZ1239 was of the Asian lineage. Comparing to the prototype strain S27, a gap of 7 aa in the nsP3 gene and missing of one repeated sequence element (RSE) in the 3’ UTR were observed in SZ1239. The E1-A226V mutation was not detected in both strains. SZ1050 and SZ1239 could infect most of the evaluated mammalian epithelial cells. The K562 cells were permissive for both SZ1050 and SZ1239 while the U937 cells were refractory to both viruses. For *Aedes* cell lines C6/36 and Aag-2, both SZ1050 and SZ1239 were able to infect and replicate efficiently.

**Conclusions:**

Compared to the prototype S27 virus, some deletions and mutations were found in the genomes of SZ1050 and SZ1239. Both viruses were susceptible to most evaluated epithelia or fibroblast cells and *Aedes* cell lines including C6/36 and Aag-2 in spite of marginal difference.

**Electronic supplementary material:**

The online version of this article (10.1186/s12985-018-1024-5) contains supplementary material, which is available to authorized users.

## Background

Chikungunya virus (CHIKV) is a mosquito-transmitted arbovirus belonging to the alphavirus genus of the *Togaviridae* family [1]. The major vectors of CHIKV are *Aedes aegypti* and *Aedes albopictus*. Importantly, CHIKV is the etiologic agent of chikungunya fever (CHIKF), a rheumatic-like disease typically characterized by high fever, prolonged polyarthralgia, myalgia, rash and sometimes death [2–4]. However, to date no effective vaccine or specific therapeutic is available to prevent or treat CHIKV infection [5].

CHIKV is an enveloped, spherical, positive sense, and single stranded RNA virus. The genome size of CHIKV is approximately 11.8Kb containing a 5′-methylguanylate cap and a 3′-polyadenylate tail as well as two open reading frames (ORFs). The first ORF encodes for four nonstructural proteins (nsP1 to nsP4), while the second ORF encodes for three structural proteins (C, E1 and E2) and two small peptides (E3 and 6 K) [6, 7]. Based on the E2 gene sequence, CHIKV is classified into four CHIKV lineages, including the West African (WA) lineage, the East/Central/South lineage (ECSA), the Asian, and the Indian Ocean lineage (IOL) [8, 9]. The IOL lineage was first distinguished from the ECSA lineage during an outbreak on the island of La Reunion in 2005–2006 [5]. The Asian lineage originated in Africa and experienced independent evolution for several centuries before its first outbreak in 1958 in Asia [10]. Since 2013, the Asian lineage has caused several epidemics in the Pacific islands and Americas [11, 12].

To date, the cell surface receptors for CHIKV in both mosquito cells and vertebrate cells remain incompletely understood [7]. Thus, to better understand the pathology of CHIKV infection, it is very important to confirm the cell types that CHIKV can attach to and productively infect. Previous studies found that different CHIKV lineages showed different cell tropisms in vitro [13] and pathogenesis in vivo [3]. For example, the *Aedes albopictus* cell line C6/36 was found to be significantly more permissive to the recently prevalent CHIKV isolates of the ECSA lineage than the original ROSS strain [13]. In another study, *Aedes albopictus* showed a higher disseminated infection and a more rapid transmission of the IOL lineage sooner after ingesting viral blood meal, while *Aedes aegypti* displayed a more severe infection and more rapid transmission of the Asian lineage after viral blood meal infection [12]. Suckling mice infected with a CHIKV strain of the Asian lineage showed a lower weight gain and higher mortality than mice infected with a strain of the ECSA lineage after intra-cerebral inoculation, despite displaying similar viral load in the brains [14]. Further gene expression studies found that the higher mortality caused by the Asian lineage was due to a differential gene expression profile involved in host immune response [14]. However, studies that compared the differences between the Asian lineage and the IOL lineage on cell susceptibility in mammalian and mosquito cell lines are limited.

In our previous study, two virus strains, SZ1050 and SZ1239, were successfully isolated from human serum samples using C6/36 cells. SZ1050 was isolated in 2010 and was from a patient returned from India [15]. SZ1239 was isolated in 2012 from a female traveler who had visited Indonesia [16]. Here, we cultured these two strains with BHK-21 cells and sequenced their whole viral genomes. Phylogenetic analysis indicated that SZ1050 belonged to the IOL lineage while SZ1239 was a strain of the Asian lineage. Next, we inoculated these two virus strains into a range of cells lines derived from different tissues of various hosts, including 293 (human embryonic kidney), HepG2 (human hepatocarcinoma), RD (human Rhabdomyosarcoma), HeLa (human cervical epithelial), THP-1 (peripheral blood monocytes from monocytic leukemia), K562 (human erythroleukemia), U937 (human histiocytic lymphoma), Ana-1 (the murinal celiac macrophage), BHK-21 (baby hamster kidney, fibroblast), MDCK (dog kidney epithelial), Vero (African Green Monkey Kidney), C6/36 (*Aedes albopictus*), and Aag-2 (*Aedes aegypti*). The viral RNA loads in the supernatant and cell lysate were evaluated.

## Results

### Phylogenetic analysis and molecular signatures of SZ1050 and SZ1239

**Fig. 1:**
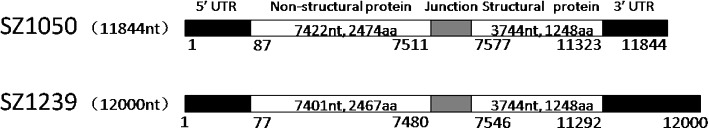
Schematic of the CHIKV isolate genomes

The complete genome sequences of SZ1050 (11,844 nt) and SZ1239 (12,000 nt) were obtained and submitted to GenBank (SZ1050: MG664850; SZ1239: MG664851). As shown in Fig. 1, the length of the structural protein was 1244 aa for both SZ1050 and SZ1239, while there was 7 aa absence in the non-structural protein of SZ1239 (2467 aa) compared to that of SZ1050 (2474 aa). In the 3’ UTR, there were three repeated sequence elements (RSEs, 35 nt) in SZ1050. Nucleotide sequence alignment revealed that there were three nucleotide differences between the first RSE (11392–11,426) of SZ1050 and that (11382–11,416) of S27 (GenBank: AF369024). There was only one nucleotide difference between the second RSE (11521–11,555) of SZ1050 and that (11525–11,559) of S27. The third RSE (11607–11,641) of SZ1050 contained the same sequence as that (11611–11,646) of S27. However, only two RSEs were observed in the 3’ UTR of SZ1239. The first RSE of S27 was absent in SZ1239. There were two nucleotide differences between the first RSE (11495–11,529) of SZ1239 and the second RSE of S27. The second RSE (11580–11,614) of SZ1239 was the same as the third RSE of S27.

**Fig. 2:**
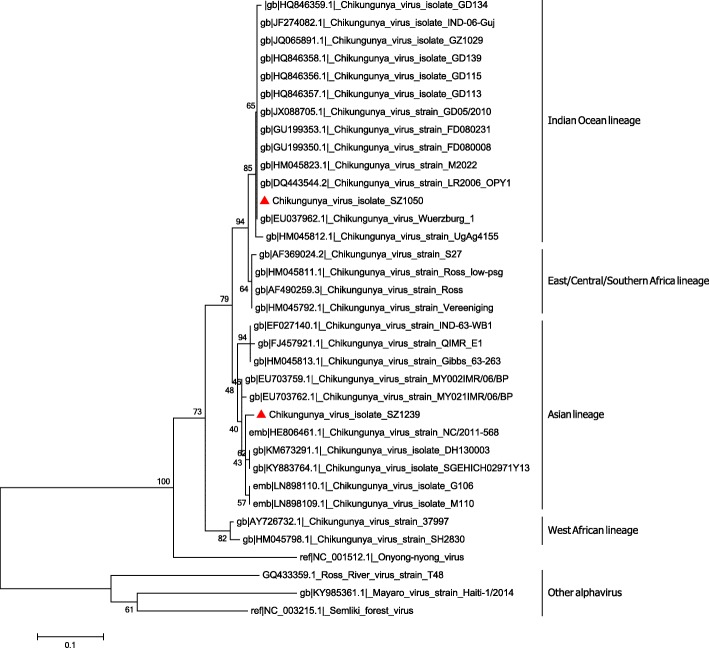
Whole genome phylogeny of chikungunya viruses and other alphaviruses. The tree illustrates the genetic clustering of available whole genomic sequences of CHIKV, ONNV, Mayaro virus (MAYV), and RRV extracted from GenBank. The evolutionary distance was inferred by using the neighbor-joining method based on the Kimura two-parameter distance model. Phylogenetic analyses were conducted in MEGA6

To classify the genotype of SZ1050 and SZ1239, phylogenetic analysis was performed based on their complete genomes as well as 33 other CHIKV sequences previously reported in the GenBank (Fig. 2). SZ1050 was observed to be in the same cluster with other CHIKV isolates of the IOL lineage [17, 18]. SZ1239 clustered with other Asian strains isolated from Indonesia and the Caribbean Island. Thus, SZ1050 belonged to the IOL lineage while SZ1239 was grouped with the Asian lineage.

On the basis of sequence analysis, the genome of SZ1050 showed the highest identity (99.94%) with that of GZ1029 (GenBank: JQ065891.1), which was identified earlier from an imported case who traveled from India to China in 2008 [19]. Compared to GZ1029, there was only one amino acid change (nsP3-X524R) and one amino acid deletion (nsP1-3P) in SZ1050. SZ1239 showed the highest identity (98.5%) with the strain DH130003 (GenBank: KM673291.1), which was isolated from a patient who returned China from Indonesia [20]. Only 4 aa and 2 aa changes were observed in the non-structural proteins (nsP2–306, nsP3–450, nsP3–517 and nsP4–100) and the structural proteins (E3–33 and E2–370), respectively.

### Amino acid differences between our viral isolates and S27

In the non-structural proteins, SZ1050 showed 32 aa changes (1.29%) compared to the African prototype strain S27 (Table 1). Most substitutions located in the nsP1 and nsP3 (0.44%). 9 out of 11 aa changes were concentrated between positions 326 and 524 in the nsP3 of SZ1050. This indicated that the fragment from 326 to 524 of nsP3 was a highly variable region, which is consistent with previous reports [20–23]. In addition, three mutations were observed in SZ1050 at relatively conserved positions: nsP2–374, nsP4–254 and nsP4–500, which were also found in certain Indian Ocean isolates [21]. In the structural protein of SZ1050, 27 aa changes (2.16%) were identified compared to S27 (Table 2). Notably, the envelop protein E2 showed the highest variation that contained 15 aa changes, significantly higher than the envelop protein E1 (0.32%) and the capsid protein (0.24%). Although E1-A226V was not found, another interesting substitution (E1-D284E) was present in SZ1050, which was also observed in some other Indian Ocean isolates [21]. E1–284 is a highly conserved position, which displays an Asp (D) in the majority of alphaviruses including CHIKV-S27, CHIKV-ROSS, o’nyong-nyong virus (ONNV), Equine Encephalitis virus (EEV), Semliki Forest virus (SFV) and Ross River virus (RRV) [21]. Further study reported that E1–284 was located on the surface of the virus particle and is involved in the contacts that make up the icosahedral E1 scaffold [21]. Whether this mutation contributes to the transmission of SZ1050 remains unclear.

**Table 1:** Amino acid mutations in SZ1239 and SZ1050 compared with S27

Protein	SZ1239	SZ1050
nsP1	P3S, P34S, L172 V, E234K,K253 M,M383 L, I384L,S454G,S473R,T478A,T481I, D486N, R491Q, L507H	T128 K, L172 V, R221S, G230R,E234K,T376 M, M383 L, I384L, T481I, Q488R,L507R
nsP2	P16L, T218S, Q273L, K338 M, M466 V, I486V, C642Y, S643 N, V756I, N768S	S14 N,H374Y,S643 N,A793V
nsP3	V166I, M213 V,Y217H, S283 N, P326S, Q332R, A334V, T336 M, V339A, K342E, I343T,377–383 gap T413 V,L434Q,V437A, M449I, Q452R,T459 V,N483D, E484D, R524G,	V175I,Y217H, P326S, V331A, T337I, A383T, I377T, K352E,L460P, S461 N, P471S
nsP4	A43L, M58 T, R85K, S90A, I101V, Y107H, Q235R, K271R, E280D, T366A, I514T, V555I,V582A, V604I	I75V, T254A, I514T, Q500L,V555I, V604I
C	Q37K, A55V, Q78R, T81 M,A93V	P23S,V27I,K63R
E3	T23I, S44R, R60H	I23T,V42I,P59S
E2	I2T, H5N, K57G, M74I, G79E,S118G, K149R,V157A, N160 T, L181 M,G205D,N207S, I211T, L248S,I255V,M267R,S299 N,Q307R, V317I, V318R, A344T,V370A,M384 V	G57 K, I74M,G79E,N160 T, A164I, L181 M, S194G, I211T,V264A, M267R, S299 N,T312 M, A344T, S375 T,V386A
6 K	T45 M,A47T, M52 L, I54V	V8I,I54V
E1	N72S,A98T, T145A, K211E, A225S, P304S, A321T, V322A, L397P	K211E, M269 V, D284E, V322A

**Table 2:** Summary of cytopathic effect induced by two CHIKV isolates at 72 h post infection

Abbreviations	Cell type	CHIKV isolates	
		SZ1050	SZ1239
HeLa	Human cervical epithelial cells	–	–
RD	Rhabdomyosarcoma	+	+
HepG2	Human hepatocarcinoma,epithelial cells	+	+
293	Human embryonic kidney, epithelial cells	+	+
K562	Human erythroleukemia line	–	–
U937	Human monocyte from histiocytic lymphoma	–	–
THP-1	Human monocytes from monocytic leuemia	–	–
Ana-1	Murinal celiac macrophage	–	–
BHK-21	Baby hamster kidney, fibroblast	+	+
MDCK	Madin-Darby canine kidney	–	–
Vero	African green monkey kidney	+	+
C6/36	*Aedes albopictus*	+	+
Aag-2	*Aedes aegypti*	–	–

‘+’ means positive for CPE, ‘-’ means negative for CPE

The amino acid variations between SZ1239 and S27 were more prominent than those between SZ1050 and S27. As illustrated in Tables 1, 67 (2.71%) and 44 (3.53%) aa changes were identified in the non-structural proteins and structural proteins, respectively. Similar to SZ1050, E1-A226V was also not detected in SZ1239 and the mutation E2-I211T was present in both strains. E1-A98T in the fusion loop of the E1 protein (E1: 83–100 aa) was detected in SZ1239 compared with that of S27. Mutation E2-I211T was present in both strains. Most of mutations were observed in the nsP3 (29 out of 67, 1.17%) and envelop protein E2 (23 out of 44, 1.84%). Of note, there was a gap in the nsP3 of SZ1239 containing 7 amino acids (HTLPSAT, 1710–1716 in the nsP3 of S27) compared to S27. Interestingly, this gap was also found in several other CHIKV isolates of Asian lineage emerged in Indonesia recently (e.g. JMB-154, GenBank: KX097982.1, 2015; DH130003, GenBank: KM673291.1, 2013).

### Mammalian epithelial and fibroblast cells were susceptible to SZ1050 and SZ1239 infection

**Fig. 3:**
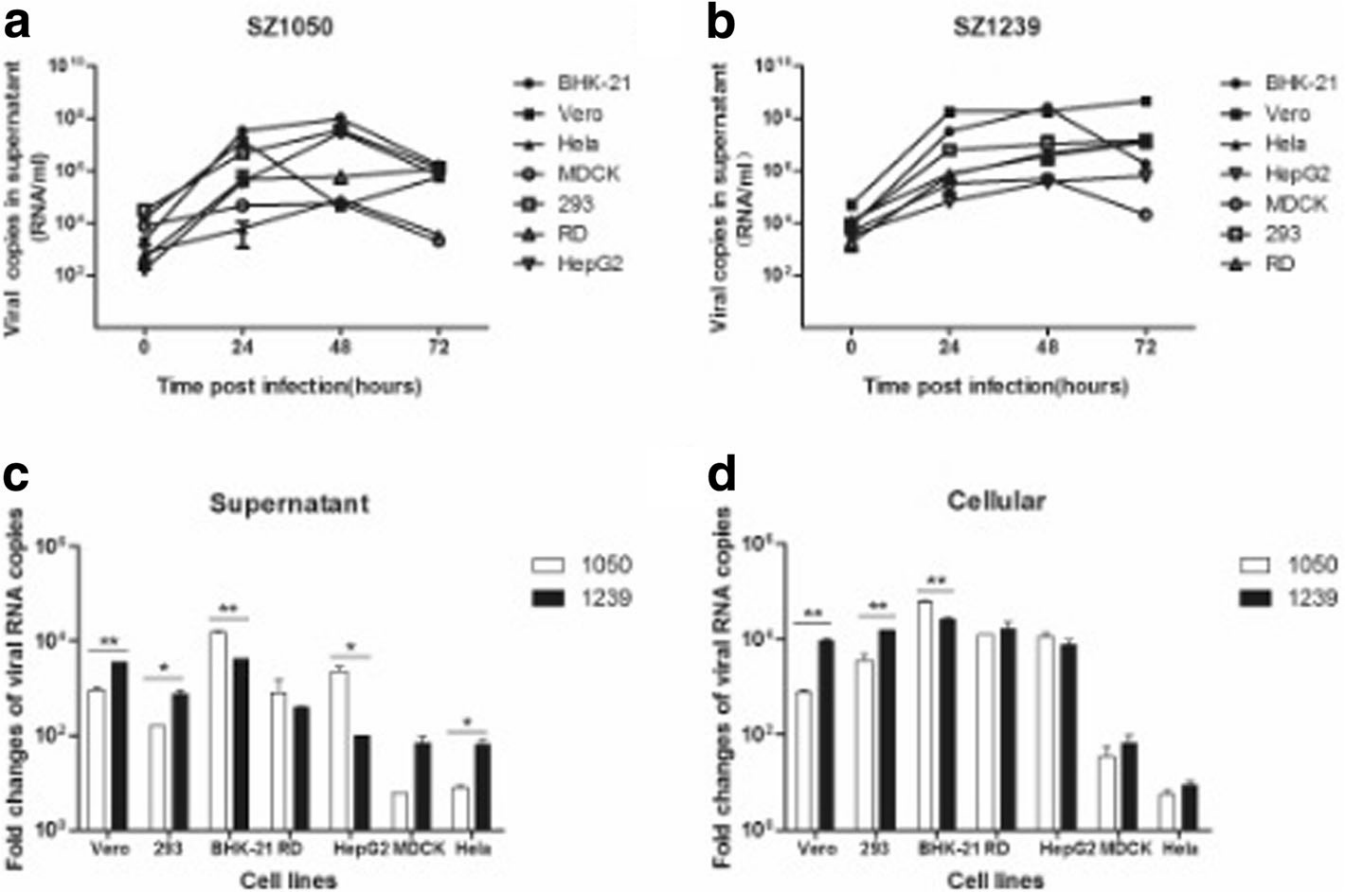
Epithelial or fibroblast cells are differently susceptible to chikungunya virus infection. (**a**) Quantification of the viral RNA load by real-time qRT-PCR from the supernatants infected with SZ1050 with MOI of 0.1 at 0, 24, 48 and 72 h. (**b**) Quantification of the viral RNA load by real-time qRT-PCR from the supernatants infected with SZ1239 with MOI of 0.1 at 0, 24, 48 and 72 h. (**c**) Comparison of viral increasing fold in the supernatant between SZ1050 and SZ1239 at 24 h. p. i. (**d**) Comparison of viral increasing fold in infected cells between SZ1050 and SZ1239 at 24 h. p. i

To investigate the differences in cell tropism between SZ1050 and SZ1239, we inoculated the two virus isolates into seven mammalian epithelial cells. Both SZ1050 (Fig. 3a) and SZ1239 (Fig. 3b) demonstrated a significant increase in viral RNA in the supernatants of BHK-21, Vero, RD, HepG2 and 293 at 24 h post inoculation. As shown in Fig. 3c, viral RNA load was increased by more than 100-folds for both SZ1239 and SZ1050. SZ1239 replicated more efficiently than SZ1050 in Vero (*p* = 0.0013), 293 (*p* = 0.0321) and HeLa cells (*p* = 0.045), while SZ1050 replicated better than SZ1239 in BHK-21 as suggested by the higher viral RNA detected in both the supernatant (Fig. 3c) and cell lysate (Fig. 3d). The replication of both viruses appeared to be less efficient in MDCK and HeLa cell lines in comparison to the other epithelial cells (Fig. 3c&d and Additional file 1: Figure S2). The typical CHIKV-specific cytopathic effects (CPE) (shrinkage, fusion, apoptosis and shedding) were obvious in BHK-21, Vero, RD and 293 cells (Table 2).

### SZ1050 and SZ1239 could produce infective virus particles in K562

**Fig. 4:**
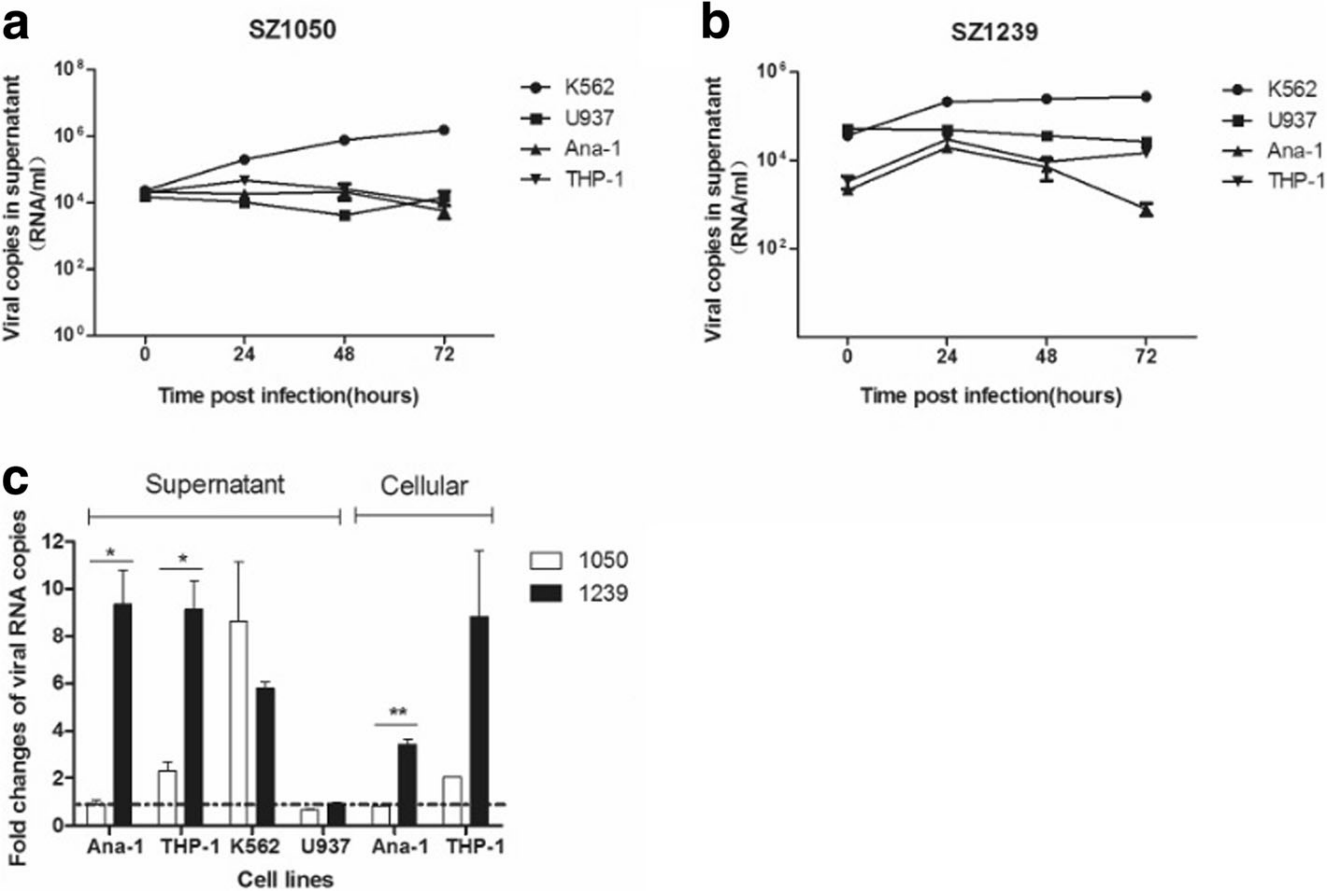
Suspension cells are differently susceptible to chikungunya virus infection. (**a**) Quantification of the viral RNA load by real-time qRT-PCR from the supernatants infected with SZ1050 MOI of 5 at 0, 24, 48 and 72 h. (**b**) Quantification of the viral RNA load by real-time qRT-PCR from the supernatants infected with SZ1239 MOI of 5 at 0, 24, 48 and 72 h. (**c**) Comparison of viral RNA increasing folds in the supernatant and infected cells between SZ1050 and SZ1239 at 24 h. p. i

To test the differences between SZ1050 and SZ1239 in cell tropism in blood cells, we selected three monocyte cell lines, K562, U937 and THP-1, as well as a macrophage cell line, Ana-1. Our data suggested that SZ1050 and SZ1239 could replicate in K562 cells (Fig. 4a and Additional file 1: Figure S3). However, both viruses failed to establish a productive infection in U937, Ana-1, and THP-1 cells (Fig. 4a&b, and Additional file 1: Figure S3). Unlike the obvious CPE in epithelial cells, no significant morphological changes were observed in these suspension cells (Table 2).

### Viral replication in mosquito cells

**Fig. 5:**
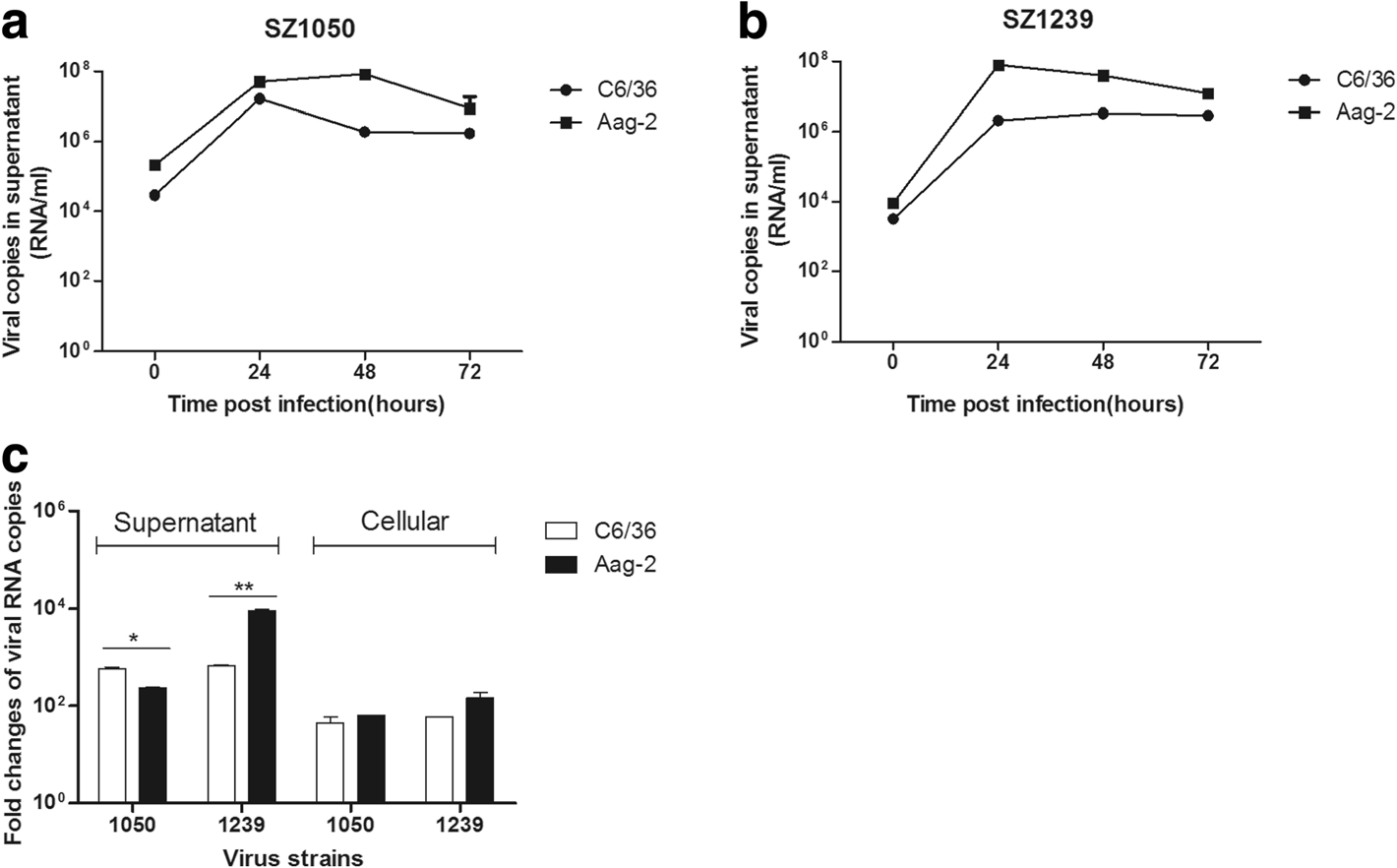
Mosquito cells are susceptible to chikungunya virus infection. (**a**) Quantification of the viral RNA load by real-time qRT-PCR from the supernatants infected with SZ1050 MOI of 0.1 at 0, 24, 48 and 72 h. (**b**) Quantification of the viral RNA load by real-time qRT-PCR from the supernatants infected with SZ1239 MOI of 0.1 at 0, 24, 48 and 72 h. (**c**) Comparison of viral RNA increasing folds in the supernatants and infected cells between SZ1050 and SZ1239 at 24 h. p. i

Since the vectors of chikungunya virus include *Aedes aegypti* and *Aedes albopictus* [24], we selected *Aedes aegypti* cell line Aag-2 and *Aedes albopictus* cell line C6/36 as mosquito cell models to investigate the cell tropisms of the two CHIKV isolates. Our results showed that both cell lines were susceptible to viral infection. The viral RNA copies were significantly increased at 24 h.p.i. (Fig. 5a&b), similar to the trend observed in the epithelial cells. Through comparing the increasing folds after infection at 24 h, we found that SZ1050 showed a higher viral RNA increase in C6/36 than that of Aag-2, while SZ1239 displayed more rapid viral RNA increase in the supernatant of Aag-2 than in that of C6/36 (Fig. 5c). Of note, virus infected C6/36 exhibited cell shrinkage and apoptosis, but no significant CPE was observed in Aag-2 (Table 2).

KY435477.1minimal changes between timepointsAag-2 embryonic *A. aegypti* CCL-125 larvae originated.

## Discussion

From the 1960s to1980s, CHIKV outbreaks were limited to Africa and Asia. In 2004, it re-emerged in Kenya and rapidly spread to several islands in the Indian Ocean as well as many other regions, including South Asia, Central and West Africa, Europe, the Caribbean and Central, South and North America served to refocus global attention to this virus [25]. Characterizing the whole viral genome and cell tropism contributes to better understand the pathogenesis and vector competence of CHIKV.

Based on the sequence analysis, we found that SZ1050 was phylogenetically most related to GZ1029, another reported CHIKV strain of IOL lineage isolated from an imported foreign case travelled from India to China in 2010 [19]. This suggested that SZ1050 was potentially a circulating CHIKV strain in India in 2010. On the other hand, SZ1239 was indicated to belong to the Asian lineage. The highest genetic identity was found between SZ1239 and another strain DH130003 of the Asian lineage, which was isolated in 2012 from a patient who traveled back to Bali from Indonesia [20]. CHIKV from the Asian lineage was the major causative agent for the increased CHIKV-infected cases in Indonesia in 2008, 2009 and 2011 [23]. Therefore, the SZ1239 strain also represented a recently circulating isolate of the Asian lineage. More recently, CHIKV of Asian lineage was also found in the pacific region and America [22, 25], indicating the Asian lineage as the major prevalent genotype of the current CHIKV outbreak.

Of note, the first RSE in S27 was deleted in the 3’ UTR of SZ1239. Similar gene gap was also found in other three CHIKV isolates from Indonesia (JMB-154, DH130003 and JMB-230), suggesting the absence of the first RSE might be due to an evolution process of CHIKV. It was reported that RSE regulated viral RNA synthesis [7]. A deletion of RSEs in model alphaviruses might affect the interaction of unknown cellular proteins involved in virus production and/or tissue specificity and leads to a reduced and delayed viral release in different cell types [26]. The consequence of the deletion of the first RSE in SZ1239 is currently unknown.

Compared to the amino acid sequences of S27, most variations in both SZ1050 and SZ1239 were observed in the nsP3 and the E2 protein (Table 1) in line with other studies [17]. The mutations in nsP3 were focused in 326–524 aa, which was a variable region [27, 28]. A gap of seven amino acids (1710–1716 in S27, 377–383 aa in the nsP3) located in the nsP3 of SZ1239 compared to S27 (Table 1). This gap should not be an occasional deletion caused by viral culture in vitro because it was also observed in an isolate in 2006 (MY/06/37350,GenBank: FN295484, Malaysia) and many recent circulating isolates of the Asian lineage such as NC/2011–568 (GenBank: HE806461.1, 2011, New Caledonia) [29], DH130003 (GenBank: KM673291.1, 2013, Indonesia) [20] and JMB-154 (GenBank: KX097982.1, 2015, Indonesia) [30]. More interestingly, compared to S27, there was a small gap of four amino acids (LPSA, 1712–1715 in S27) in the middle of this seven amino acid-gap in the nsP3 of some CHIKV isolates found in Micronesia: Yap State in 2013 (strain 3807,GenBank: KJ451622.1, 2013) [31] and America (isolate 14.02217, GenBank: KY435477.1, 2014) [22]. However, there was no gap for the CHIKV isolates identified in Malaysia in 2007 or before (strain MY002IMR/06/BP, GenBank: EU703759.1; strain MY003IMR/06/BP, GenBank: EU703760.1; strain M125, GenBank: KM923917.1). This suggested that the four amino acids (1712–1715) might play a key role in the evolution of CHIKV.

It was reported that the C-terminal hypervariable domain of nsP3 (398–406 aa) prevented stress granule formation through sequestration of GTPase-activating protein (SH3 domain)-binding proteins (G3BPs) during the mammalian stress response [32]. Depletion of G3BPs caused severely reduced levels of negative-stranded (and consequently also positive-stranded) RNA [32]. Fross et al. also identified the hypervariable C-terminal domain (475–501 aa) of nsP3 as a critical factor for granular localization and sequestration of mosquito Rin (G3BP homologue Rin in live mosquitoes) [33]. The 18 amino acid deletion in nsP3 (386–403 aa) in Sindbis virus strain AR86 affected neurovirulence in mice [34]. Whether the gap (377–383 aa) in the nsP3 of SZ1239 would affect the interaction between G3BPs and nsP3 or contribute to the neurovirulence requires further investigation.

In addition, the E1-A226V mutation, which was reported to relate to the adaption in *Aedes albopictus* [35], was not detected in SZ1050 and SZ1239. In the previous two small-scale outbreaks in China in 2010, E1-226 V was observed in all four isolated CHIKV strains [18]. Therefore, the possibility of the SZ1050- or SZ1239-induced CHIKV outbreak in China would be low because *Aedes albopictus* is the major vector responsible for arbovirus transmission in most Chinese regions. Interestingly, another mutation, I211T, was found in E2 of both SZ1050 and SZ1239, which was also found in the West African lineage and Asian lineage [10]. Virus containing E1-A226V and E2-I211T showed enhanced infectivity of CHIKV in *Aedes albopictus* [36]. Whether the single E2-I211T mutation could contribute to the transmission advantages of the virus in *Aedes* remains largely unkown.

To further investigate the cell tropism of SZ1050 and SZ1239, we inoculated two viral isolates in thirteen cell lines. The viral RNA increasing speed in the first 24 h in RD, HepG2 and 293 was significantly higher than that in HeLa cells (Fig. 3d), which suggested that CHIKV virus might prefer to infect and replicate in human liver, kidney and muscle rather than in the cervix. This could partially explain why many patients showed symptoms of myalgia and dysfunction of liver.

Beside adherent cells, suspension blood cells were also detected to compare their susceptibility to these two CHIKV strains. Our results showed that U937 cells were refractory to CHIKV infection in agreement with other reports [5, 13, 24]. A previous study found that CHIKV was able to bind to a cell membrane protein–prohibitins in U937 cells but could not replicate in them [37]. The detailed mechanism is unclear. Similarly, our result showed that Ana-1 was refractory to infection by SZ1050 from the IOL lineage. In addition, we also infected PMA-stimulated THP-1 with SZ1050 and SZ1239. Although SZ1239 showed higher viral RNA copies in the supernatant than that of SZ1050, it was hard to confirm that it could effectively replicate in THP-1 due to the minimal viral RNA load changes between time points. It was reported that CHIKV (La Reunion isolate of IOL lineage) could neither bind THP-1 at 4 °C nor produce infective viruses at 37 °C [24]. More studies should be performed to investigate the cell susceptibility for CHIKV infection in unstimulated THP-1 and PMA-stimulated THP-1 cells.

As CHIKV is an arbovirus, it is very important to evaluate the virus tropism in the mosquito vectors. In this study, *Aedes aegypti* cell line Aag-2 and *Aedes albopictus* cell line C6/36 were selected to serve as two mosquito cell models. Our results suggested that both cell lines were susceptible to these two strains. Of note, in our study, Aag-2 cells were very susceptible to both CHIKV isolates but had no CPE after infection.

This was not consistent with the findings of another study [13]. In that study, the authors found chikungunya virus of the ECSA lineage not able to effectively infect the *Aedes aegypti* cell line CCL-125 cells [13]. Possible explanation might come from the differences on the sources and contaminated pathogens between the two *Aedes aegypti* cell lines (Aag-2 and CCL-125). On one hand, Aag-2 was derived from embryonic *A. aegypti* while CCL-125 was larvae originated; on the other hand, Aag-2 was contaminated with Phasi charoen-like virus (PCLV) and Cell-fusing agent virus (CFAV), while CCL-125 was onlyinfected with PCLV [38]. The single infection of CFAV was believed to promote the infection of DENV [39]. Therefore, it is possible that CFAV in Aag-2 can modulate the infection of CHIKV.

## Conclusions

To the best of our knowledge, this is the first study to explore the differences of the genome characters and cell tropisms between CHIKV strains of the Asian lineage and the IOL lineage. We found some mutations and gaps in our viral genomes compared to the sequence of S27. Both viruses could efficiently replicate in most evaluated epithelia or fibroblast cells and two *Aedes* cell lines. Our findings provided valuable information to better understand the pathogenesis and vector competence for current circulating CHIKV lineages.

## Methods

### Cells and culture conditions

BHK-21 (ATCC® CCL-10™), HepG2 (ATCC® HB-8065™), Hela (ATCC® CCL-2™), RD (ATCC® CCL-136™), MDCK (ATCC® CCL-34™), THP-1 (ATCC® TIB-202™), U937 (ATCC® CRL-1593.2™), K562 (ATCC® CCL-243™), 293 (ATCC® CRL-1573™) and C6/36 (ATCC® CRL-1660™) were obtained from ATCC (American Type Culture collection, Manassas, VA). Ana-1 and Vero cell were kept in our laboratory. The *Aedes aegypti* cell line Aag-2 was kindly provided by Professor Gong Cheng, Tsinghua-Peking Center for Life Sciences, School of Medicine, Tsinghua University, China. BHK-21, HepG2, 293, Vero, MDCK, Hela and RD cells were cultured at 37 °C, 5%CO_2_ in Dulbecco’s modified Eagle’s medium (DMEM, Gibco, Invitrogen) supplemented with 10% heat-inactivated fetal bovine serum (HIFBS; Gibco, Invitrogen) and 100 units of penicillin and 100μg streptomycin/ml (1%P/S). C6/36 cells were cultured at 28 °C, 5%CO_2_ in DMEM supplemented with 10% HIFBS and 1%P/S as described previously [40]. Aag-2 cells were cultured at 28 °C, 5%CO_2_ in Schneider’s Drosophila medium supplemented with 10% HIFBS and 1% P/S in line with other study [41]. Cytopathic effects (CPE) were examined at 24, 48 and 72 h.p.i. with invert light microscopy.

### Viral isolate and propagation

Virus was cultured in BHK-21 cells for three times. The supernatant were harvested three days after infection when CPE was evident. Cell debris was clarified by centrifugation, and virus was stored at − 80 °C in single-use aliquots. Virus stock titers were determined by standard plaque assay in Vero cells, and expressed as plaque-forming units (PFU/ml).

### RNA extraction and quantitative reverse transcription PCR (qRT-PCR)

Viral RNA was extracted from supernatant with a QIAamp Viral RNA Mini kit (QIAGEN, Germany) according to the manufacturer’s recommended procedures. Briefly, 140 μl of each sample was first treated with 560 μl of AVL buffer containing 10 μg/ml of carrier RNA, followed by alcohol precipitations. The precipitations were then applied onto the QIAamp Mini columns and the viral nucleic acids were absorbed onto the silica-gel membrane after centrifugation. Finally, the viral pellet was resolved in 50 μl of RNase-free water. Viral RNA copies were quantified using qRT-PCR, as described previously [1].

### Sequence and phylogenetic analysis

Viral first strand cDNA was obtained by using the ReverTra Ace qPCR RT Kit (TOYOBO, Japan). The nucleotide sequence of the S27 strain was used for primer designing (GenBank accession no. AF369024) [42]. The whole genome was sequenced by PCR and Sanger sequencing. Amplification was achieved using an AccuPrime™ Taq DNA Polymerase, High Fidelity (12346–086, Invitrogen). The 5’ UTR and 3’ UTR were sequenced by using the SMARTer RACE 5′/3’ Kit (TAKARA, Japan). All the sequences were then assembled with DNAMAN 5.2.2.

Phylogenetic analysis based on the available full-genome sequences of CHIKV was performed by using MEGA version 6.0 [43]. For the construction of phylogenetic trees, the neighbor-joining algorithm and the Kimura two-parameter distance model were utilized. The reliability of the analysis was evaluated by a bootstrap test with 1000 replications.

### Virus growth curves

To detect the cell susceptibility of both strains, seven mammalian adherent epithelial or fibroblast cells were infected with both SZ1050 and SZ1239 in 24-well plates at a multiplicity of infection (MOI) of 0.1PFU/cell. After incubation with virus for 1 h at 37 °C, cells were washed for three times with 1XPBS, and replaced with 1 ml maintaining medium (DMEM+ 2%HIFBS+ 1%P/S). Then, cells were further incubated at 37 °C, 5% CO_2_ for three days.

Similarly, four suspension blood cells were infected with both CHIKV strains in 24-well plates at a MOI of 5 PFU/cell. Cells were resuspended in RPMI 1640 medium supplemented with 2% FBS and 1% P/S after incubation and washing. Two *Aedes* cell lines C6/36 and Aag-2 were also inoculated with two CHIKV isolates and cultured at 28 °C, 5% CO_2_ for three days. Supernatants and infected cells were harvested at 0, 24, 48 and 72 h.p.i. by RT-QPCR and plaque assay, as described previously. The primers for QPCR were designed in the conserved N-terminal of nsP3 (108-146aa). CHIKV-F: TCCTCTCCACAGGTGTATACTCAGG (4398–4422 in S27), CHIKV-R: CTTGTCTCGGCAGTAGATGACCAC (4490–4513 in S27).

### Standard plaque assay

To determine the titer of infectious virus in the supernatant, we performed standard plaque assay as described previously [13]. Briefly, Vero cells were seeded in 6-well plate the day before test. Next day, when the cell confluence reached up to approximately 100%, cells were inoculated with ten-fold diluted virus. After incubation for 1 h at 37 °C with constant agitation, the supernatant was discarded, and DMEM medium supplemented with 2% HIFBS and 1% low melting point gel was added to each well. Plates were incubated for further 48–72 h at 37 °C. Then the wells were fixed and stained. Plaques were counted by naked eyes. Each experiment was done independently in triplicate.

### Statistical analysis

The data of viral load were expressed as mean ± SD. For the statistical analysis of two groups’ viral load increasing folds, a Student unpaired *t-*test was done. The level of statistical significance was set at *p* < 0.05. Degrees of significance are indicated in the figure caption as follow:* *p* < 0.05; ** *p* < 0.01; ***p < 0.001. All experiments were repeated three times.

## Additional file

Additional file 1: **Figure S1.** CPE of virus infected cells at 72 h.p.i. **Figure S2.** Viral titer in the supernatant of virus infected adherent cells by plaque assay. **Figure S3.** Viral titer in the supernatant of virus infected suspension cells by plaque assay. (DOCX 3689 kb)

## Abbreviations

CFAV: Cell-fusing agent virus
CHIKV: chikungunya virus
CPE: cytopathic effects
DMEM: Dulbecco’s modified Eagle’s medium
ECSA: East/Central/South Lineage
EEV: Equine Encephalitis virus
HIFBS: heat-inactivated fetal bovine serum
IOL: Indian Ocean lineage
MOI: multiplicity of infection
ONNV: O′ nyong-nyong virus
P/S: penicillin and streptomycin
PCLV: Phasi charoen-like virus
PFU: plaque-forming units
qRT-PCR: quantitative reverse transcription PCR
RRV: Ross River virus
RSEs: repeated sequence elements
SFV: Semliki Forest virus
UTR: untranslated repeat region
