# Supplementary material for: Differences in genome characters and cell tropisms between two chikungunya isolates of Asian lineage and Indian Ocean lineage
Source: Virol J. 2018 Aug 20;15:130. doi: 10.1186/s12985-018-1024-5 (PMC6102929; doi:10.1186/s12985-018-1024-5)
Supplement: Supplementary file 1 — Figure S1. CPE of virus infected cells at 72 h.p.i. Figure S2. Viral titer in the supernatant of virus infected adherent cells by plaque assay. Figure S3. Viral titer in the supernatant of virus infected suspension cells by plaque assay. (DOCX 3689 kb) [file 12985_2018_1024_MOESM1_ESM.docx]

Vero BHK-21


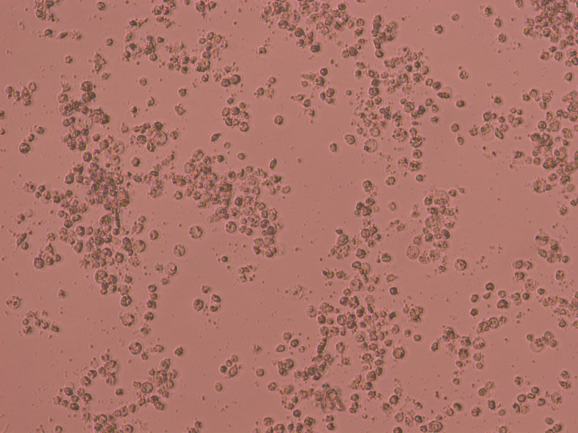

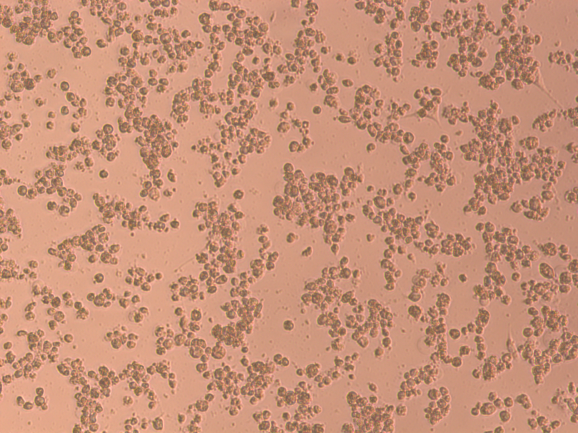


293 RD


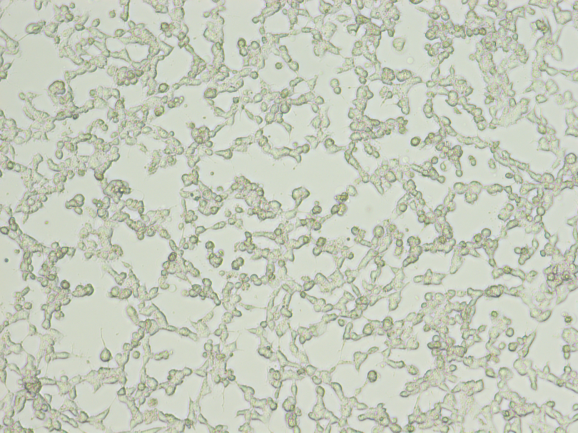

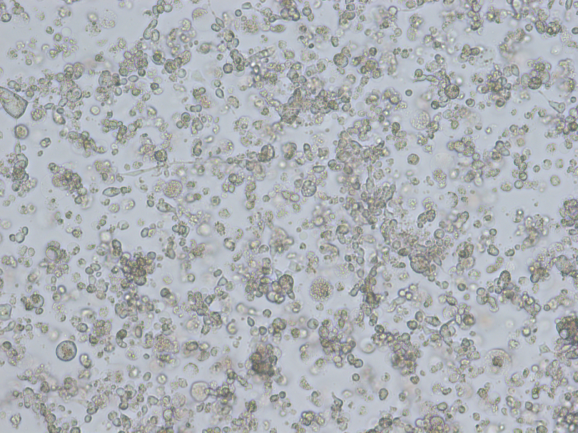


C6/36


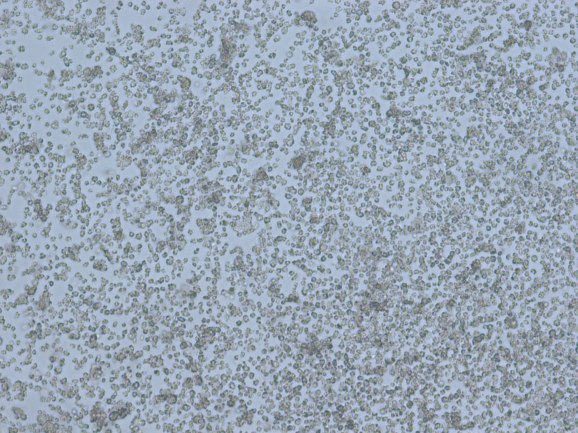


FigS1 CPE of virus infected cells at 72h.p.i.


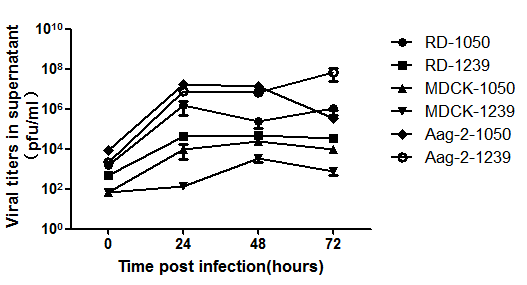


Fig S2 Viral titer in the supernatant of virus infected adherent cells by plaque assay.


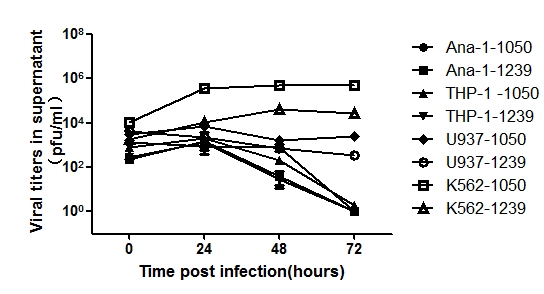


FigS3 Viral titer in the supernatant of virus infected suspension cells by plaque assay.
